# Case report: A successful case of targeted immunotherapy for locally advanced pancreatic cancer under non-surgical conditions

**DOI:** 10.3389/fimmu.2024.1519186

**Published:** 2025-01-10

**Authors:** Yuanbo Bi, Haotian Yv, Xiaopeng Ma, Shengxiong Chen

**Affiliations:** ^1^ Department of Hepatobiliary Surgery, The Second Hospital of Hebei Medical University, Shijiazhuang, Hebei, China; ^2^ Department of Oncology, Hebei Medical University, Shijiazhuang, Hebei, China

**Keywords:** locally advanced pancreatic cancer, targeted immunotherapy, immune checkpoint inhibitors, camrelizumab, tumor-associated macrophages

## Abstract

**Introduction:**

Locally advanced pancreatic cancer (LAPC) is a borderline unresectable malignancy that presents significant treatment challenges. The management of LAPC remains a complex issue, particularly in patients who are not eligible for surgical resection.

**Case:**

Here, we report the case of a 60-year-old woman diagnosed with LAPC through pathological biopsy who subsequently underwent targeted immunotherapy following the failure of a gemcitabine, oxaliplatin, and S-1 (G&S) chemotherapy regimen.

**Intervention:**

Based on next-generation sequencing (NGS), the patient’s treatment regimen was adjusted to include albumin-bound paclitaxel and capecitabine chemotherapy, along with the PD-1 inhibitor camrelizumab (200 mg/cycle) for six cycles. Throughout the treatment period, the patient consistently declined surgical intervention. Imaging studies, including an upper abdominal computed tomography (CT), revealed the formation of a calcified layer surrounding the cancerous tissue in the pancreatic head. Remarkably, the patient has shown stable disease and no evidence of metastasis since the initiation of targeted immunotherapy.

**Conclusion:**

This case highlights the potential of targeted immunotherapy for the treatment of LAPC, particularly in non-surgical patients. A personalized approach guided by NGS, combined with immunotherapy, is an effective alternative to traditional treatment strategies for managing this challenging malignancy.

## 1 Introduction

Pancreatic cancer is projected to become the second leading cause of cancer-related deaths by 2030, with only 15% of patients presenting with resectable disease at diagnosis (1). Early-stage pancreatic cancer often presents with mild gastrointestinal symptoms such as nausea, vomiting, abdominal distension, and low back pain. Consequently, many patients are misdiagnosed and treated for other conditions, leading to delays in the appropriate management of pancreatic cancer (2–4). Surgical resection remains the gold standard for achieving a radical cure; however, for borderline resectable pancreatic cancer, R0 resection following chemoradiotherapy is considered superior to the treatment for locally advanced pancreatic cancer (LAPC). According to the DPCG criteria, LAPC is defined as involving >90° arterial encasement (such as the superior mesenteric artery, celiac trunk, or any hepatic artery) and/or >270° involvement or occlusion of the portal vein and/or superior mesenteric vein (5). Palliative chemotherapy (with or without radiotherapy) and supportive care are typically the primary treatment options for patients with LAPC (6).

The standard treatment for LAPC remains a subject of debate internationally. The most recent clinical guidelines, including the 2024 REDISCOVER guidelines, “Conversion Surgery” position paper from the joint meeting of the International Association of Pancreatology, and Japan Pancreas Society in 2022, suggest that in cases of favorable response to neoadjuvant chemotherapy, surgical resection can be considered following discussion with the patient and their family. However, both guidelines emphasize the lack of sufficient evidence to recommend a specific timeline for surgical resection after neoadjuvant chemotherapy (7, 8).

Current treatment options for LAPC are evolving, with recent studies exploring the combination of stereotactic body radiotherapy (SBRT), chemotherapy, and immune checkpoint inhibitors (ICIs). Notably, the combination of FOLFIRINOX (FFX) chemotherapy followed by SBRT has shown the potential for improving survival outcomes in patients with LAPC, especially in selected individuals. This approach may also increase the likelihood of radical resection in patients whose tumors were initially deemed unresectable (9). Additionally, ICIs targeting CTLA-4, PD-1, and PD-L1 have demonstrated superior efficacy compared to cytotoxic chemotherapy alone (10, 11). However, both strategies are still in the clinical research phase, and further studies are required to validate these findings.

McCarthy et al. reported a case involving a patient with locally advanced pancreatic ductal adenocarcinoma who underwent treatment with PD-1 inhibitors and radiotherapy, followed by surgical resection, achieving nearly complete pathological remission after surgery (10). In contrast, we present a case of chemotherapy combined with targeted immunotherapy, without radiotherapy or surgical resection, which resulted in a favorable prognosis. We also reviewed the current literature for reports on the use of targeted immunotherapy for the treatment of pancreatic cancer, highlighting its potential role in improving outcomes in patients with LAPC.

## 2 Case presentation

**Figure 1:**
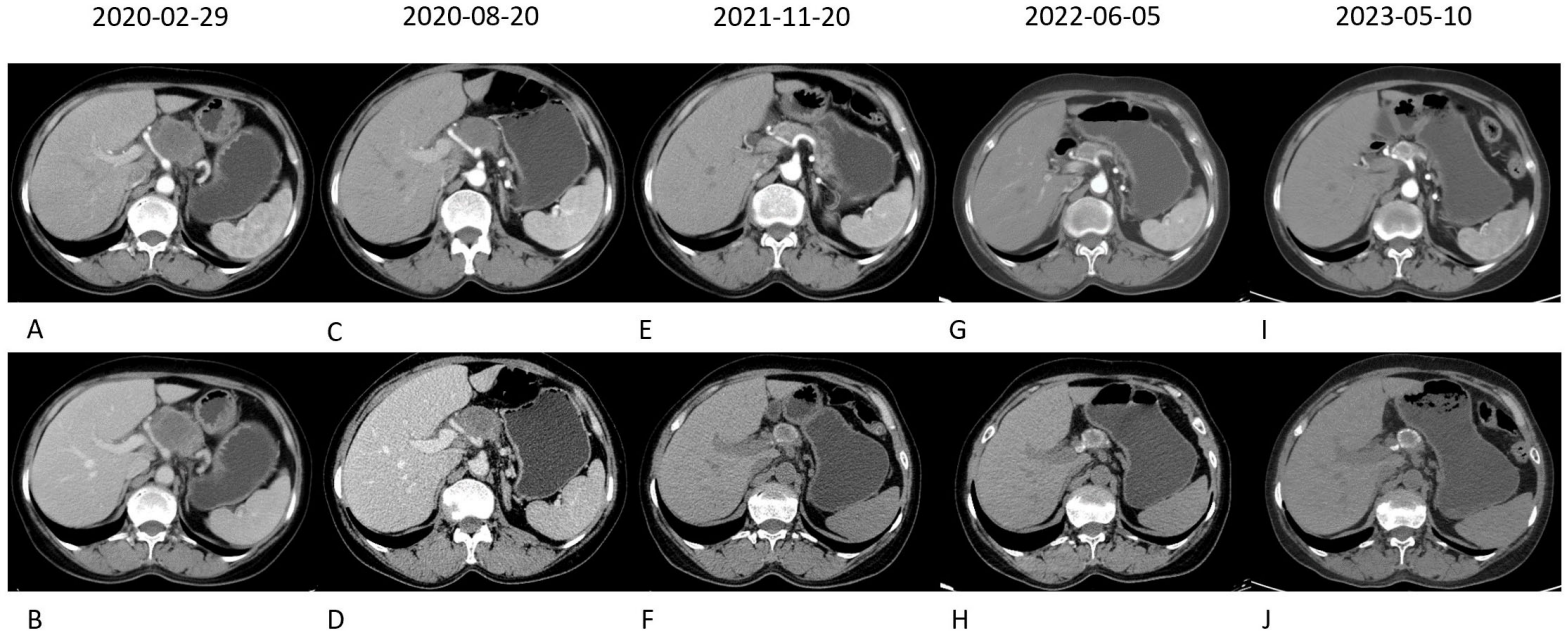
Contrast-enhanced plain CT showed the mass in close contact with the surrounding blood vessels **(A, B)**. After 2 months of targeted immunotherapy, the tumor was slightly reduced, and the relationship with the surrounding blood vessels was clearer **(C, D)**. After conservative treatment for more than 1 year, the mass was significantly reduced and a ring of high-density shadow appeared around it, which was considered to form a calcification package **(E–H)**. The size of the mass remained the same, whereas the density of the annular calcification layer increased **(I, J)**.

A 60-year-old female presented with a history of intermittent upper abdominal pain and discomfort lasting >10 d. Contrast-enhanced CT of the upper abdomen revealed a mass in the pancreatic neck with dilatation of the pancreatic duct, suggestive of a tumor (**Figure 1**).

### 2.1 Auxiliary examinations

**Figure 2:**
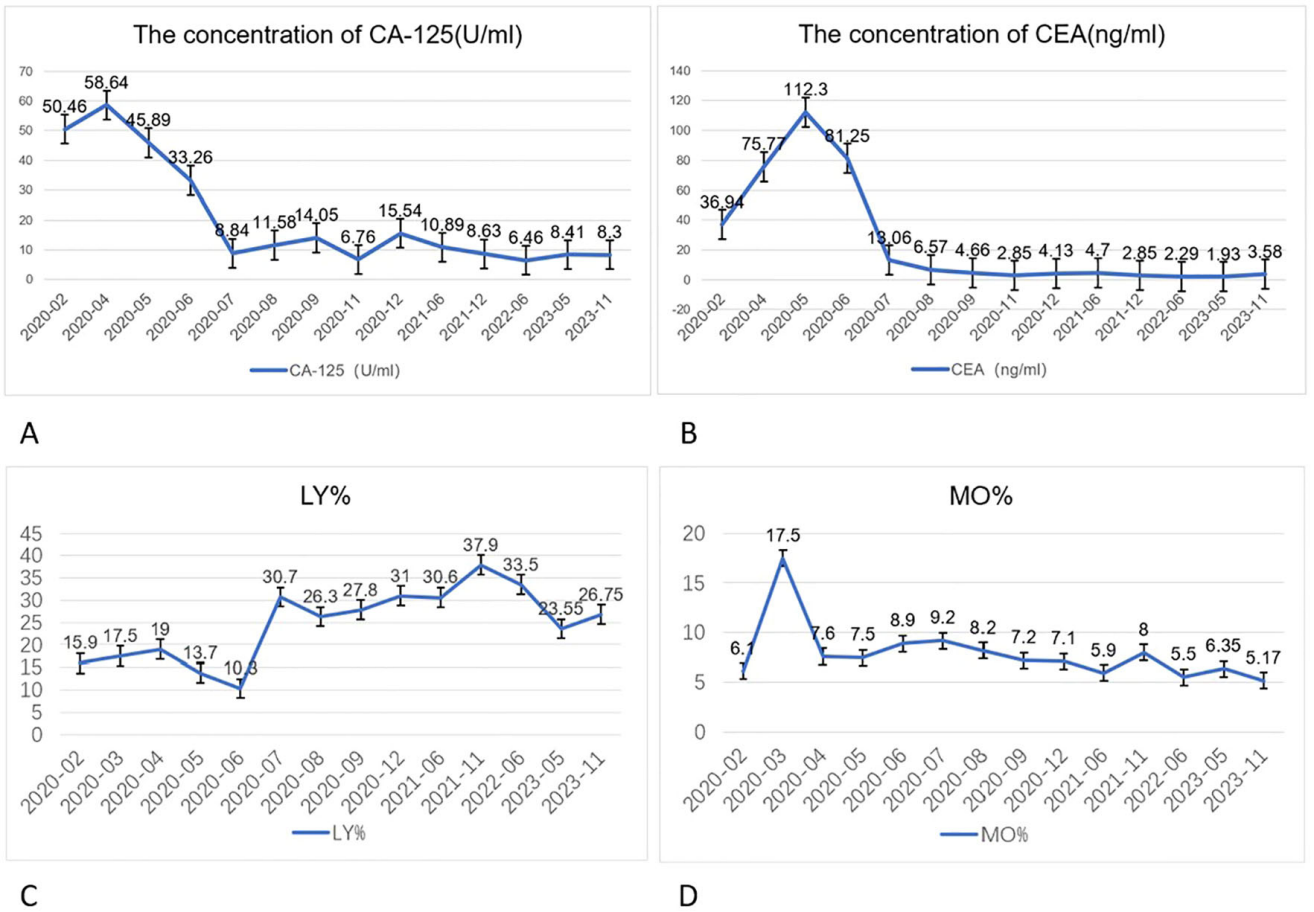
Changes in CA125 **(A)** (reference range: 0–35) and CEA **(B)** (reference range: 0–10) levels in the patient before and after treatment. These tumor markers showed a brief elevation in the initial treatment stage, followed by a decrease below the baseline level. Lymphocyte percentage (LY%) **(C)** returned to normal after three cycles of targeted immunotherapy (reference range: 20.00–50.00 %), and peripheral monocyte percentage (MO%) **(D)** returned to normal after the first cycle of targeted immunotherapy (reference range: 3.00–10.00 %).

Routine blood tests, liver function tests, electrolyte levels, coagulation profiles, and preoperative evaluation revealed no significant abnormalities. However, serum tumor marker levels were elevated: carcinoembryonic antigen (CEA), 36.94 ng/mL (reference range: 0–10 ng/mL) and cancer antigen 125 (CA125), 50.46 U/mL (reference range: 0–35 U/mL). The patient’s lymphocyte percentage (LY%) was 15.9% (reference range: 20–50%), and the peripheral monocyte percentage (MO%) was 6.1% (reference range: 3–10%) (**Figure 2**).

Endoscopic ultrasound-guided fine-needle aspiration confirmed LAPC. Genetic testing revealed and mutation rates of 8.82% and 6.17% for the *KRAS* and *TP53*, respectively. Additionally, PD-L1 mRNA expression was moderately positive (28%).

### 2.2 Initial treatment

The patient initially received a chemotherapy regimen consisting of gemcitabine, oxaliplatin, and S-1 (G&S regimen) for three cycles, along with oral pain management using acetaminophen and oxycodone tablets. Follow-up CT of the upper abdomen revealed a slightly enlarged pancreatic neck mass with persistent pancreatic duct dilatation. Serum CEA levels continued to rise, indicating a limited response to the initial chemotherapy.

### 2.3 Treatment modification

The treatment regimen was modified based on the results of genetic testing. The treatment was switched to a combination of albumin-bound paclitaxel and capecitabine, along with the PD-1 inhibitor camrelizumab (200 mg, administered for six cycles). After three cycles of this new regimen, the patient developed multiple skin hemangiomas, necessitating the addition of apatinib. However, apatinib induced hypertension, which was treated with lenvatinib.

### 2.4 Further response and follow-up

The patient refused surgery throughout treatment. Follow-up imaging via enhanced CT showed that the pancreatic head mass had developed a high-density shadow at its periphery, suggesting the formation of a calcified layer surrounding the tumor. There was no evidence of metastasis to surrounding tissues or organs. Tumor marker levels gradually returned to normal, and the patient’s condition stabilized.

## 3 Outcome

Forty-five months after the diagnosis, the patient’s serum CEA level decreased to 3.58 ng/mL, and the CA125 level was reduced to 8.30 U/mL. Treatment was discontinued, and there has been no evidence of recurrence.

This case highlights the potential of targeted immunotherapy combined with chemotherapy in the treatment of LAPC, particularly in patients who are not candidates for surgical resection.

## 4 Discussion

**Figure 3:**
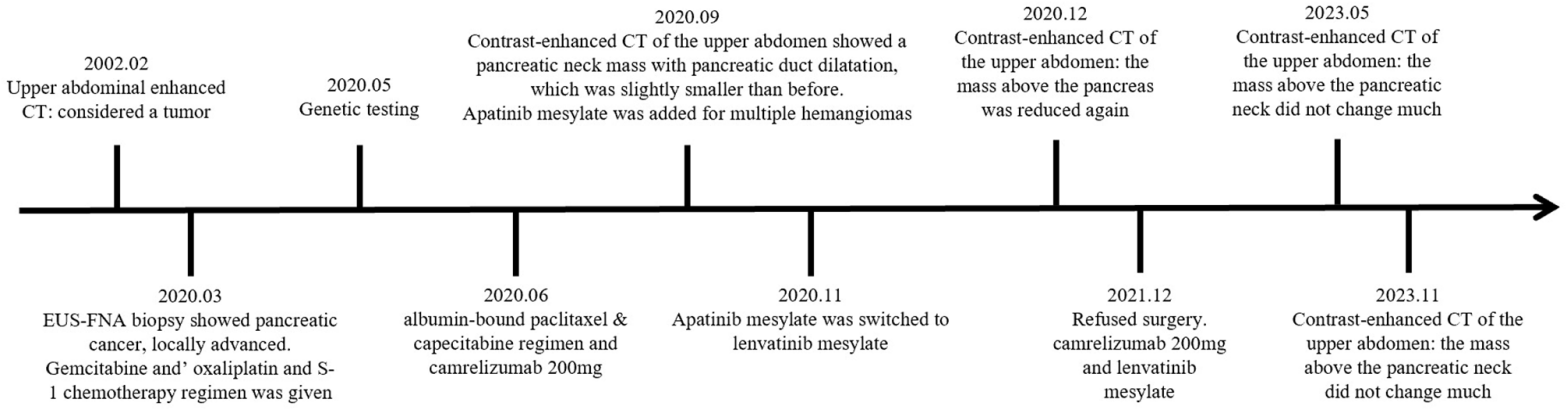
Timeline of the patient’s treatment.

Herein, we presented the case of a 60-year-old woman with LAPC who initially received ineffective chemotherapy (G&S regimen). Genetic testing revealed mutations in *KRAS* (mutation rate: 8.82%) and *TP53* (mutation rate: 6.17%). Subsequently, the patient was treated with the PD-1 inhibitor camrelizumab, which led to a remarkable outcome, with complete remission after 45 months of treatment, despite the patient’s consistent refusal to undergo surgery (**Figure 3**).

The management approach for this case aligns with the American Society of Clinical Oncology Clinical Practice Guidelines, which suggest that targeted therapies such as PD-1 inhibitors could be considered in cases of LAPC with specific genetic alterations (12). After six cycles of targeted immunotherapy, an in-hospital discussion concluded that the patient had reached resectable conditions, leading to a recommendation for surgical resection. This is consistent with the latest clinical guidelines, which emphasize the potential utility of baseline CEA and CA125 in monitoring patients with LAPC. In this case, both tumor markers normalized after just three cycles of targeted immunotherapy.

Interestingly, the MO% decreased to normal levels after the first cycle of targeted immunotherapy, while the LY% returned to normal after three cycles. Monocytes have long been considered cytotoxic to various tumor cells and are often used as a prognostic marker in cancer, reflecting the tumor microenvironment (TME). The peripheral monocyte count correlates with the density of tumor-associated macrophages (TAMs), which play a critical role in immune evasion. In colorectal and pancreatic cancer, TAMs are known to suppress immune responses (13, 14). TAMs can be regulated by Treg cells, which prevent T-cell activation and inhibit the immune response via the fatty acid synthesis pathway mediated by sterol regulatory element-binding protein 1 (15, 16). The reduction in monocyte levels in this case may indicate a favorable modulation of the TME following immunotherapy.

Recent studies have suggested that patients with *KRAS* mutations tend to respond poorly to chemotherapy combined with immunotherapy. The *KRAS* G12D mutation induces immunosuppressive tumor environments in the early stages of malignancy, partly by expanding and reprogramming cancer-associated fibroblasts (CAFs) (17, 18). The deletion of CAFs has been shown to restore T-cell effector function and reduce tumor proliferation (19). However, in this case, the combination of albumin-bound paclitaxel, capecitabine chemotherapy, and camrelizumab demonstrated an unexpectedly favorable effect on the patient’s prognosis despite the presence of *KRAS* mutations.

Camrelizumab is a humanized PD-1 inhibitor that blocks the interaction between PD-L1 and its receptor PD-1, preventing immune evasion by tumors. In pancreatic cancer, PD-L1 expression is closely associated with CD163+ TAMs, and the cytokine TNF-α significantly increases PD-L1 mRNA expression by activating NF-κB signaling (20–22). Camrelizumab has demonstrated positive results in liver and advanced gastric cancer (23, 24). Furthermore, studies have shown that combining PD-1 inhibitors with neoadjuvant chemoradiotherapy improves the LAPC resection rate without significantly increasing postoperative complications (25). Animal experiments have shown that combined immunotherapy targeting TNFR2 and PD-1/PD-L1 signaling can regulate PD-L1 expression at the transcriptional level through the p65 NF-κB pathway, induce the activation of CD8+ T cells in the pancreatic ductal adenocarcinoma (PDAC) microenvironment, and induce strong anti-tumor immune memory and secondary prevention to achieve better therapeutic effects (26). Other studies have shown that third-generation PD-L1-targeted CAR T cells enhance T cell persistence (27). In this case, after treatment with camrelizumab, follow-up CT revealed a significant reduction in the pancreatic mass, making surgical resection a feasible option.

Despite the development of multiple hemangiomas that were manageable with medication, the patient’s overall response to the targeted immunotherapy regimen was promising. This case highlights the potential of targeted immunotherapy, particularly in patients with LAPC who are not candidates for surgery or show inadequate responses to conventional chemotherapy. These results suggest that further research on targeted immunotherapy for LAPC is warranted, especially for patients with high PD-L1 expression, or those who do not tolerate or respond to cytotoxic chemotherapy.

The traditional treatment for pancreatic cancer mainly relies on surgery, chemotherapy, and radiation therapy. Only 15% of pancreatic cancer cases are considered resectable at the time of diagnosis. A retrospective study showed that only 4.3% of the 413 patients who received FFX or gemcitabine plus albumin-bound paclitaxel treatment successfully underwent surgery, with a median overall survival (OS) of 32.9 months (2, 28). In a study of induction chemotherapy combined with stereotactic body radiotherapy, the local recurrence rate of patients with LAPC who underwent conversion therapy for surgical resection was as high as 33% (29). One study showed that the median OS of 54 patients treated with proton beam therapy was 18.2 months, with only one patient surviving for >5 years (30). Mustafa Suker et al. showed that the median OS for patients undergoing FFX therapy was 24.2 months (95% CI 21.7–26.8) (31). A recent single-center retrospective cohort study showed that the median OS of patients with advanced pancreatic cancer treated with a sequence of FFX and gemcitabine/albumin-bound paclitaxel was 10.3 months (32). Passardi et al. followed up on 73 patients who received gemcitabine and oxaliplatin and large fractionated stereotactic radiotherapy for LAPC and showed that the median progression-free survival and OS were 10.2 (95% CI 7.8–13.2) and 14.3 (95% CI 12.0–18.1) months, respectively (33). A study that included 1047 patients who underwent LAPC radiofrequency ablation (RFA) showed that the median OS ranged from 6 to 33 months (34). Compared to traditional treatments, targeted immunotherapy may bring about a new era in the treatment of pancreatic cancer, especially unresectable pancreatic cancer. There are few large-sample prognostic studies on targeted immunotherapy for LAPC, and successful cases without surgery are even rarer. The significant effects observed in this case may promote the development of targeted immunotherapy and individualized treatments for pancreatic cancer.

Additionally, this case did not consider the costs or accessibility of the treatments, nor did it explore alternative therapies. Furthermore, the relationship between ICIs and TAMs warrants further investigation, as these interactions may provide new therapeutic opportunities.

## 5 Literature review

A literature search was conducted using PubMed, Scopus, Web of Science, JAMA Network, and NEJM to identify cases in which targeted immunotherapy was successfully used to treat pancreatic cancer. Among the cases reviewed, one patient with PDAC underwent surgical resection after a combination of radiotherapy and targeted immunotherapy. Another case involved a patient with pancreatic head cancer and liver metastasis, who received targeted immunotherapy combined with RFA. Another case involved a patient with pancreatic acinar cell carcinoma, who had enlarged retroperitoneal lymph nodes and multiple pulmonary nodules. The patient responded well to targeted immunotherapy after chemotherapy failure. In all three cases, the patients tolerated the treatment well and showed no evidence of disease recurrence (**Table 1**).

**Table 1:** Literature review.

Author	Age (years)	Sex	Diagnosis	Study title	Intervention measures	Result
McCarthy PM, Rendo MJ, Uy MD, et al (10)	83	Female	Pancreatic ductal adenocarcinoma (PDAC)	Near Complete Pathologic Response to PD-1 Inhibitor and Radiotherapy in a Patient with Locally Advanced Pancreatic Ductal Adenocarcinoma	Stereotactic body radiotherapy (SBRT) and pembrolizumab and surgery	Tolerate therapy well without evidence of disease recurrence
Zhu Y, Ning Z, Meng Z (35)	49	Female	Pancreatic head cancer with liver metastases	Case Report: Overcoming challenges in pancreatic cancer with liver metastases: a personalized therapeutic odyssey of TACE, ablation, and immunotherapy	TACE therapy with gemcitabine and cisplatin and microwave ablation	Yielding satisfying therapeutic outcomes with commendable tolerability
Xu H, Wang X, Zhou S, Hu Q, Cao D (36)	68	Male	Pancreatic acinar cell carcinoma (PACC) with retroperitoneal enlarged lymph nodes and multiple nodules in both lungs	Efficacy of chemotherapy combined with toripalimab in PD-L1–positive and high tumor mutation burden pancreatic acinar cell carcinoma: case report	Two cycles of gemcitabine and nab-paclitaxel and two cycles of SOX regimen and toripalimab and SOX regimen	Satisfactory response and tolerance

In summary, the treatment method in this case has met the requirements of the latest international guidelines. However, due to the small sample size and high heterogeneity, our treatment method is not universally applicable to patients. However, this case report contributes to the growing body of evidence supporting the use of targeted immunotherapy in LAPC, particularly when combined with chemotherapy, without the need for radiotherapy or surgical resection. The favorable prognosis in this case provides valuable insights into potential treatment strategies for patients with LAPC.

## 6 Conclusion

In the present case, targeted immunotherapy led to a rare phenomenon in which the pancreatic head tumor was encased in a calcified layer encapsulating the cancerous tissue. Despite recent studies indicating that patients with *KRAS* mutations often respond poorly to chemotherapy combined with immunotherapy, this case highlights an unexpected and favorable outcome. The combination of albumin-bound paclitaxel, capecitabine chemotherapy, and camrelizumab significantly improved patient prognosis, demonstrating the potential of targeted immunotherapy in treating LAPC.

The traditional treatment for pancreatic cancer relies primarily on surgery, chemotherapy, and radiation therapy. This case highlights the promising efficacy of ICIs in LAPC, offering hope to patients with advanced unresectable disease. However, further research is needed to define the specific criteria for patient selection, as the clinical application of ICIs in LAPC remains complex and individualized. The remarkable results observed in this case may help advance targeted immunotherapy and personalized treatment for pancreatic cancer. Targeted immunotherapy should be considered a viable treatment option for frail or unresectable LAPCs.

## Data Availability

The original contributions presented in this study are included in the article/supplementary materials. For further inquiries, please contact the corresponding author.
